# Supplementary material for: Assessment of Raman Spectroscopy for Reducing Unnecessary Biopsies for Melanoma Screening
Source: Molecules. 2020 Jun 20;25(12):2852. doi: 10.3390/molecules25122852 (PMC7355922; doi:10.3390/molecules25122852)
Supplement: Supplementary file 1 [file molecules-25-02852-s001.pdf]

molecules

Supplementary Materials: The following are available online, Figure S1: Representative Raman spectra of melanoma and pigmented lesions, Figure S2: Receiver Operating Characteristic (ROC) curve for classifying melanoma vs. pigmented lesions with k-fold cross-validation (k=4, stratified), Table S1: Detailed clinical data summary, Table S2: Summary of lesion information.

Because Raman scattering is inherently a weak scattering process, we have to trade off acquisition time for a good signal to noise ratio (SNR). We have attempted to strike the right balance in these measurements and achieved adequate SNRs for integration times of 2 seconds [1,2]. We have found that these SNRs are sufficient for the extraction of discriminating biophysical signals [3,4] and are consistent with what other groups have reported [5–8].

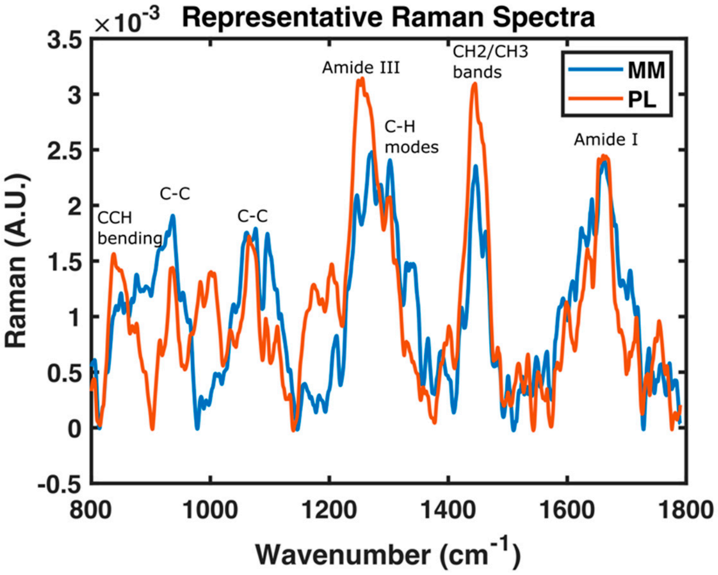

Figure S1: Representative Raman spectra of melanoma and pigmented lesions

In addition to leave-one-out cross validation, k-fold cross validation (k=4) with stratified sampling was applied. Briefly, the original sample was randomly partitioned into 4 equal-sized subsamples for melanoma (MM) and pigmented lesions (PL) respectively, and each of the 4 subsamples of MM and PL combined was used exactly once as the validation data. Note that the subset of PCs was selected within the cross-validation loop, so the calculated PCs might not be the same between leave-one-out and k-fold cross validation strategies and could be slightly different in different loops within a cross validation strategy. With k-fold cross-validation (k=4), the area under the ROC curve (AUROC) of 0.884 means high accuracy in distinguishing melanoma from pigmented lesions. If our recommendations based on Raman spectroscopy had been enacted, approximately 73.6% of the biopsies on pigmented lesions could have been avoided while accurately detecting all melanoma lesions in the data set (sensitivity of 100%). The blue shade shows the 95% confidence interval for the ROC. We think that the k=4 (stratified) cross-validation approach is less desirable than leave-one-out cross-validation which provides the maximum number of melanoma training cases in each iteration, and the comparable performance between both cross-validations shows the stability of our model.

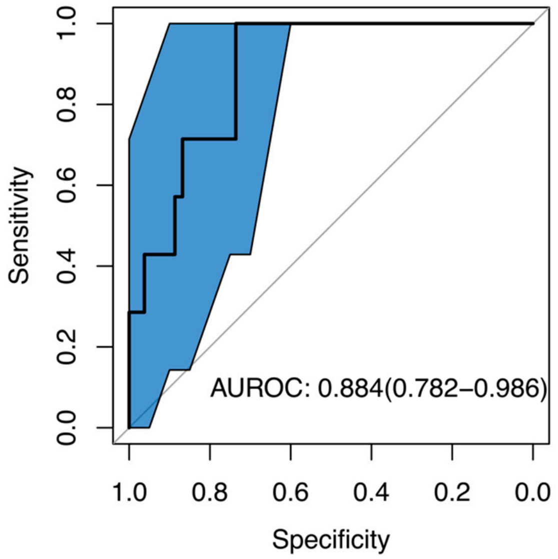

Figure S2. Receiver Operating Characteristic (ROC) curve for classifying melanoma vs. pigmented lesions with k-fold cross-validation (k=4, stratified). The area under the ROC curve (AUROC) of 0.884 means high accuracy in distinguishing melanoma from pigmented lesions. If our recommendations based on Raman spectroscopy had been enacted, approximately 73.6% of the biopsies on pigmented lesions could have been avoided while accurately detecting all melanoma lesions in the data set (sensitivity of 100%). The blue shade shows the 95% confidence interval for the ROC.

Table S1. Detailed clinical data summary.

**Lesion type**	**# Patients (n=52)**	**# Lesions (n=60)**	**# Measurements (n=185)**
**Pigmented lesions**	**46**	**53**	**158**
Benign nevus	18	21	59
Mildly dysplastic nevus	17	21	64
Moderately dysplastic nevus	6	6	19
Severely dysplastic nevus	5	5	19
**Melanoma**	**6**	**7**	**27**

Table S2. Summary of lesion information.

**Final lesion diagnosis**		**Benign nevus**	**Mild. dys. nevus**	**Mod. dys. nevus**	**Sev. dys. nevus**	**MM**
Subjects	Mean age (range)	41 (18-72)	45 (24-75)	51 (35-71)	42 (19-81)	53 (28-87)
	Male	8	11	6	3	4
	Female	13	10	0	2	3
Number of lesions		21	21	6	5	7
Number biopsied (%)		21 (100%)	21 (100%)	6 (100%)	5 (100%)	7 (100%)
Location	Head and Head	2	2	1	0	3
	Trunk	12	16	5	3	3
	Upper limb	6	1	0	0	1
	Lower limb	1	2	0	2	0

Abbreviations: Mild. dys. nevus: mildly dysplastic nevus; Mod. dys. nevus: moderately dysplastic nevus; Sev. dys. nevus: severely dysplastic nevus; MM: Melanoma

References

1. Moy, A.J.; Feng, X.; Markey, M.K.; Reichenberg, J.S.; Tunnell, J.W. Noninvasive skin cancer diagnosis using multimodal optical spectroscopy. In Proceedings of the Photonic Therapeutics and Diagnostics XII; International Society for Optics and Photonics, 2016; Vol. 9689, p. 968905.
2. Moy, A.J.; Feng, X.; Nguyen, H.T.M.; Zhang, Y.; Sebastian, K.R.; Reichenberg, J.S.; Tunnell, J.W. Spectral biopsy for skin cancer diagnosis: initial clinical results. In Proceedings of the Photonics in Dermatology and Plastic Surgery; International Society for Optics and Photonics, 2017; Vol. 10037, p. 1003704.
3. Feng, X.; Moy, A.J.; Nguyen, H.T.M.; Zhang, Y.; Zhang, J.; Fox, M.C.; Sebastian, K.R.; Reichenberg, J.S.; Markey, M.K.; Tunnell, J.W. Raman biophysical markers in skin cancer diagnosis. *JBO* **2018**, *23*, 057002, doi:10.1117/1.JBO.23.5.057002.
4. Sharma, M.; Marple, E.; Reichenberg, J.; Tunnell, J.W. Design and characterization of a novel multimodal fiber-optic probe and spectroscopy system for skin cancer applications. *Review of Scientific Instruments* **2014**, *85*, 083101, doi:10.1063/1.4890199.
5. Lui, H.; Zhao, J.; McLean, D.; Zeng, H. Real-time Raman spectroscopy for in vivo skin cancer diagnosis. *Cancer Res.* **2012**, *72*, 2491–2500, doi:10.1158/0008-5472.CAN-11-4061.
6. Jermyn, M.; Mercier, J.; Aubertin, K.; Desroches, J.; Urmey, K.; Karamchandiani, J.; Marple, E.; Guiot, M.-C.; Leblond, F.; Petrecca, K. Highly Accurate Detection of Cancer In Situ with Intraoperative, Label-Free, Multimodal Optical Spectroscopy. *Cancer Res* **2017**, *77*, 3942–3950, doi:10.1158/0008-5472.CAN-17-0668.
7. Desroches, J.; Jermyn, M.; Pinto, M.; Picot, F.; Tremblay, M.-A.; Obaid, S.; Marple, E.; Urmey, K.; Trudel, D.; Soulez, G.; et al. A new method using Raman spectroscopy for in vivo targeted brain cancer tissue biopsy. *Scientific Reports* **2018**, *8*, 1792, doi:10.1038/s41598-018-20233-3.
8. Haka, A.S.; Shafer-Peltier, K.E.; Fitzmaurice, M.; Crowe, J.; Dasari, R.R.; Feld, M.S. Diagnosing breast cancer by using Raman spectroscopy. *PNAS* **2005**, *102*, 12371–12376, doi:10.1073/pnas.0501390102.

© 2020 by the authors. Licensee MDPI, Basel, Switzerland. This article is an open access article distributed under the terms and conditions of the Creative Commons Attribution (CC BY) license (http://creativecommons.org/licenses/by/4.0/).
